# New Approach to Analysis of Selected Measurement and Monitoring Systems Solutions in Ship Technology

**DOI:** 10.3390/s19081775

**Published:** 2019-04-13

**Authors:** Boleslaw Dudojc, Janusz Mindykowski

**Affiliations:** Gdynia Maritime University, Department of Marine Electrical Power Engineering, ul. Morska 81-87, 81-225 Gdynia, Poland; j.mindykowski@we.umg.edu.pl

**Keywords:** ship systems, measurement, monitoring, main engine parameters, temperature measurement, ship environment, hazardous areas

## Abstract

This paper is dedicated to certain types of measurement in ship systems, analyzed based on selected case studies. In the introductory part, a simplified structure of a modern cargo ship as an object of measurement and control is presented. Next, the role of measurement in the ship’s operation process is described and commented on, with focus on specifics of local and remote control, both manual and automatic. The key part of the paper is dedicated to a short overview of selected examples of measuring and monitoring systems. The basic criteria for the aforementioned selection are the vital role of the considered systems for safe and effective ship operation as well as documented innovative contribution of Gdynia Maritime University (GMU) in development of the state-of-the-art in the analysed area of measurement. Based on these criteria, the monitoring of operational parameters of main engine and temperature measurement in the ships hazardous areas have been chosen. The aforementioned measurement and monitoring systems are analysed, taking into account both innovation of technical solutions together with their ship technology environment conditions and related legal requirements. Finally, some concluding remarks are formulated.

## 1. Introduction

The core information of this paper is based on a recently published paper [1], describing the problem of the vital role of measurement and monitoring of the operational parameters of main engine (power system) and temperature measurement in the ship’s hazardous areas (cargo system) for safe and effective ship operation as a whole. 

**Figure 1:**
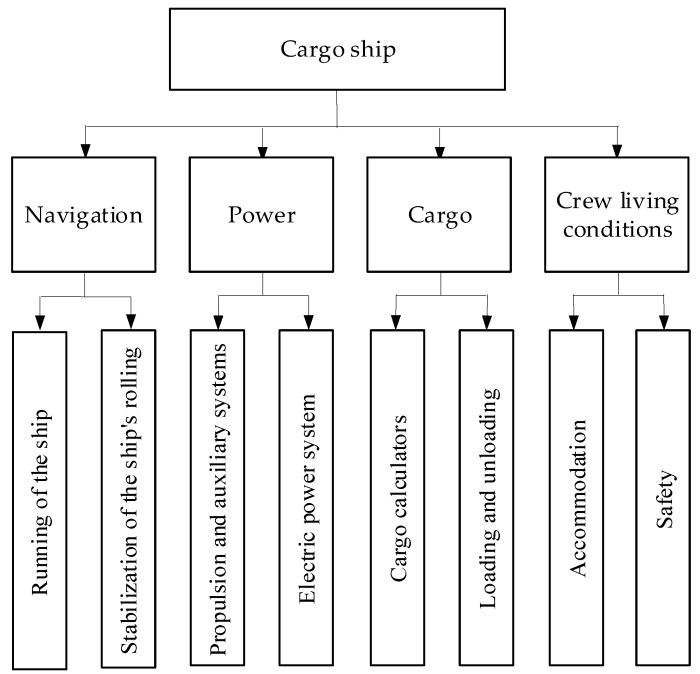
Systems and subsystems of typical cargo ship. (Updated version based on [2]).

Ships are very sophisticated maritime, industry objects. On the one hand, each ship or other floating object differs from the other, but on the other hand, all of them have a lot of common properties. A modern automated cargo ship as an object of measurement and control presents a complex structure of four main systems: navigation, power, cargo and crew living conditions. In each system there can be distinguished some subsystems, which are illustrated in Figure 1 (for each system there are only two corresponding subsystems, but there can be more). Among all those mentioned above, the electric power subsystem is very important and it has a significant influence on the other systems and subsystems. It is worth underlining that there is a close relation between electric power and propulsion systems. More and more often we can meet not only with traditional sources of electrical energy as a set of diesel-generator units for electric power, but other configurations are also used, e.g., additional shaft generators or generators supplied by system utilizing heat from exhaust gases of the main engine. The electrical generators can also be driven by classical engines powered by gas or gas turbines. In recent years, electric ships have been explored more intensively and high voltage electrical systems are often used to additionally raise efficiency of such solutions. Taking into consideration different aspects like efficiency, reliability, environmental protection and economical effectiveness, AC (Alternating Current) electrical systems are already partially supported by DC (Direct Current) creating hybrid electric power system, and in the near future they can even be replaced by these new solutions, especially on small ships using renewable energy sources.

It is worth mentioning that at the ships available, electrical power is comparable to the loads, which is automatically adjusted. Among all mentioned above, the electric power subsystem is very important and it has a significant influence on the other systems and subsystems. All of the very sophisticated systems of a ship require supervision by monitoring and control systems. Additional systems with safety functions are installed. Safety functions have higher priority than normal operation functions. 

At present, usually the distributed microprocessor systems are used for implementation of the measurement, monitoring, control and safety functions. Microprocessor systems are more susceptible to electromagnetic interference and disturbances of power supply than traditional analog systems. 

In this paper two typical case studies related to ship practice are presented. These studies are addressed to critical operational parameters, like main engine parameters in ships’ power plants or temperature in ship tanks. A novelty of this paper concerns the carrying out and analysis of the main engine signals as well as the experimental research of newly implemented SeaPerformer System—in the case of main engine research. In the second case, a new approach concerns in-depth theoretical analysis of the temperature measurement line of liquefied gas in tanks together with consequences resulting from it. This in-depth analysis is resulting in identifying and explaining the reasons of new additional error, which may appear during the temperature measurement in the ship’s hazardous areas. To indicate unexpected alternating current component, disturbing a correct temperature measurement, there was patented a new impendence simulator of Pt-100 sensors, which enable to improve significantly the quality and reliability of commissioning of the intrinsically safe measurement line under investigation Following this chapter, the rest of this article is structured in this way. Section 2 describes the role of measurement in the ships’ operation from different points of view. Section 3 is fundamental for this paper, being devoted to a short overview of selected examples of measuring and monitoring systems on sea ships, and presents, firstly, a ship performance monitoring system concept based on SeaPerformer System case study measurement of operational parameters of main engine, and finally, the closing subsection of Section 3 analyzes the temperature measurements in the ship’s hazardous areas. Section 4 summarizes the conclusions of the study, stressing proposed novelties against the state-of-the-art. 

## 2. The Role of Measurement in the Ship’s Operation Process 

To achieve complete overview of any condition on board considered sea ship, it is vital to measure not only affecting parameters such as flow, pressure, temperature and level [3,4,5], but also the operational parameters of main engine, or more widely—ship propulsion [6,7]. Special attention should be paid to the measurement systems supporting navigation area [8,9,10]. This information allows crew members to take action before changes affect equipment performance and cause downtime or failures. 

Considering the ship as a measurement and control object, there are different tasks executed in the following areas:

1. Navigation;
2. Operation of power plant;
3. Operation of cargo system;
4. Ensuring safety of crew, equipment and environment.

In the navigation area, these tasks may include:

- Measurement, filtration and estimation of ship position coordinates (measurement of the position of the ship and foreign ships) for the purpose of controlling its movement along the desired trajectory, position stabilization and use in anti-collision systems;
- Measurement of parameters characterizing movement of the ship (determining the velocity vector, course, draft, trim, side swaying of the ship, wind direction and force).

Measurement tasks for safe and economical operation of the ship’s power plant, i.e., the main engine and all the mechanisms and auxiliary devices consist of:

- Measurement of mechanical motion parameters of devices driving the generators and selected characteristic parameters, such as the quality of the fuel fed to them and the composition of exhaust gases;
- Measurement of parameters, in general temperatures, pressures, levels, viscosity and flows at selected points determining operational conditions of the power plant;
- Measurement of torque, power and revolution speed on the shaft of the Main Engine (ME) for the purpose of economical operation of the ship’s propulsion;
- Measurement of quantities characterizing the power grid (voltages, currents, powers, frequency, cos φ, insulation resistance, THD—Total Harmonic Distortion);
- Fire protection system.

Cargo transportation requires the following measurement tasks:

- Measurement of cargo parameters that determine its qualitative features;
- Measurement of the amount/weight of the transported cargo;
- Load distribution measurement (loading calculators);
- Measurement of technological media parameters enabling transport of cargo;
- Measurement of water levels inside of ballast tanks.

Qualified crew members are required to operate the ships adequately. Ships have to provide proper accommodation and additional systems necessary to support good nutrition and health of the crew while ensuring adequate environmental protection. The following measurement tasks are done in order to fulfil that:

- Measurement of parameters of potable water system;
- Measurement of parameters of air conditioning system;
- Measurement of parameters of cooling system for food storage;
- Measurement of parameters of sewage system;
- Identification of smoke and temperature of fire alarm system.

Safety function of the ship as a whole requires control of limit values and trends in their changes, basic parameters determining safe operation of technical systems and safety of people, cargo and the marine environment.

The aforementioned measuring tasks include both operational and diagnostic measurement, but the authors are of the opinion that the latter ones are beyond the main scope of this paper. The purpose of operational measurement is to determine the current value of the parameters of a given system and to check the correctness of its operation and related processes.

Operational measurement is used for both manual and automatic remote control, and its main purpose is safe and effective operation of the ship. In case of emergency, vital systems can be controlled locally, where all the system operations are done by the crew directly, sometimes using only human power. Reading of parameters is made on the instrumentation placed directly on the controlled device or machine. Local control of the main engine or local control of the rudder are examples of such a solution.

In case of remote manual control, the results of operational measurement are read from the instrumentation installed in the Main Switchboard or on the relevant Engine Control Room (ECR), Cargo Control Room (CCR) desks or Bridge Control Console (BCC) navigation bridge console. They are used in multi-parameter regulation systems, where a human factor plays a basic role. 

For the remote automatic control, the respective automatic control loops of controlled systems are used without human interference.

In both approaches presented above, the same set of measured parameters from sensors and transducers are used for systems control. Some measured parameters can be doubled or made according to the special solution to ensure adequate level of reliability. 

All measured points of measured parameters are scattered on the ship. In spite of measured parameters, a connection from a sensor or a transmitter to a measurement unit has to be done by wires. A sensor or a transmitter (field devices), connecting wires and input circuits of measurement unit create independent electrical circuits which will be named measurement lines. The lengths of used wires can be from a few to hundreds of meters. The measurement lines of measurement systems are most sensitive to influence of interferences and disturbances. Additional requirements should be done in the case of parameters measured in hazardous areas. Modification to explosion protection can limit measurement properties or even introduce additional errors.

Hundreds or sometimes more than one thousand parameters can be measured on the modern ships. Most from them are measured by binary sensors (on-off type, even around 50%), analog transmitters (two wire current 4–20 mA standard, about 20–30%) and for measurement of temperature most often thermo-resistive Pt-100 sensors (also Pt-500 or Pt-1000) or rarely thermocouple are used. Measurement of temperature is realized by connection of sensors either to the measurement unit directly or to the transmitters with standard output electrical signals. In such cases, the two wire current 4–20 mA standard is still used very often in hybrid option. Hybrid option concerns a very popular solution where both the analog and digital communication are used simultaneously. Most common available transmitters are a solution with Highway Addressable Remote Transducer (HART) Protocol, invented and introduced by Rosemount Inc. The pure digital transmitters Manchester Coding, Bus Powered (MBP) for Foundation Fields or Profibus protocols are also available as the field devices but still less popular in shipbuilding applications.

## 3. A Short Overview of Selected Examples of Measuring and Monitoring Systems on Sea Ships 

Measurement methods in marine technology have passed from the model of direct and indirect measurement into the system measurement phase. The most important characteristics of the system measurement are:

- Measurement must be treated together with the measured object,
- Measurement is accompanied by instrumentation, usually called a measuring and/or monitoring system, whose role is to implement a set of operations, among others:
  - Detection of signals from the measured object;
  - Analog-to-digital conversion of signals, which consists of sampling, quantization and digital coding;
  - Operational processing of signals, mainly digital, carried out using computer technology, consisting of forming signals carrying information about measured quantities; usually we deal with multi-path processing.

A short overview of selected examples presented below is based on the selection criteria concerning firstly a vital role of the considered system for safe and effective ship’s operation and secondly well documented contribution of Gdynia Maritime University in the development of the state-of-the-art in the analyzed area of measurement.

### 3.1. Ship Performance Monitoring System

Ship performance monitoring systems [10,11,12] are implemented on modern vessels in order to support efficient and environmentally sound operation. Such systems integrate vast amounts of signals and process them with respect to ship energy efficiency. An example of such a system is SeaPerformer developed by Research and Development Company Enamor Ltd. Gdynia, Poland, which cooperates closely with Gdynia Maritime University. SeaPeformer has been developed within the framework of research project co-financed by Polish National Centre for Research and Development (research project POIR.01.01.01-00-0933/15). The system has been successfully implemented on dozens of deep sea ships. 

SeaPerformer is a modular solution which consists of:

- Data processing and storage server based on marine industrial computer which serves the purpose of data acquisition, storage and automated processing. Server allows user interaction with the help of dedicated user interface and support ship-to-shore data exchange realized with the help of the existing satellite communication system;
- One or more data collecting units (typically there are two units, one located on the ship bridge for the purpose of navigation signals and one installed in the engine control room for collecting machinery signals);
- Power supply unit featuring system safe shut down in the case of blackout and automatic system start when power is restored;
- One or more high resolution and large size (24″ or more) marine industrial monitors with keyboard and trackball serving user interaction.

System interconnection is realized with the help of a dedicated or general ship Ethernet network. This solution simplifies system installation in the case of retrofit projects and allows data access with the use of other computers within the same LAN (Local Area Network).

**Figure 2:**
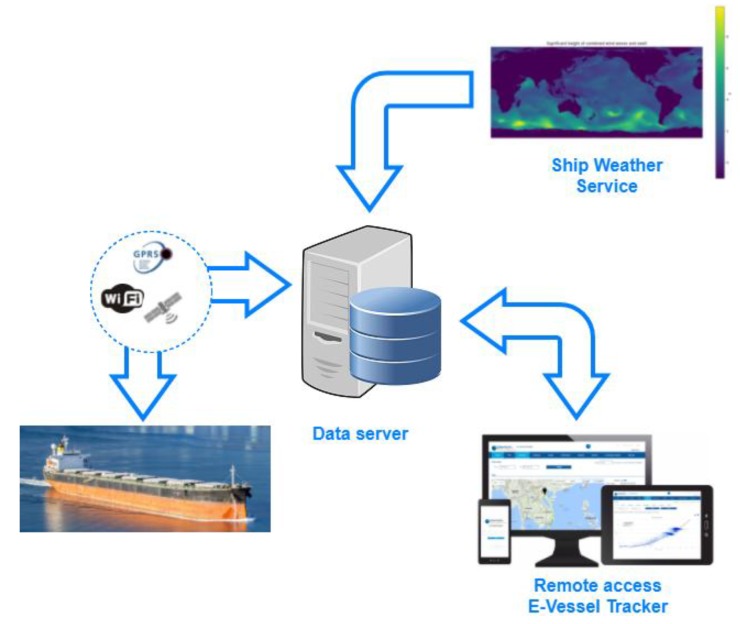
Scheme of ship to shore data transfer and online access to data with the use of web application.

Usually, such a system is capable of the simultaneous recording of a large amount of data. Depending on the type of ship, main engines and subsystems, SeaPerformer collects from a few hundred up to a couple thousand signals. Data collection is performed with the use of two general hardware interfaces: NMEA 0183 for the communication with navigation equipment such as ship GPS, log, echo-sounder, gyro-compass, weather station etc. and Modbus (RTU or TCP) for data collection from machinery e.g., main and auxiliary engines, boilers, fuel system, alarm and monitoring system, loading computer etc. Information collected with the use of the mentioned interfaces are stored in two databases. The high frequency database collects signals with frequency ranging from 1 Hz up to 100 Hz as appropriate, with respect to the nature of the signal. Due to the large amount of information, this database is kept onboard and can be transferred onshore only on request, usually when detailed signal examination is needed for the purpose of troubleshooting. Standard ship-to-shore data exchange is facilitated with used low frequency database created by 1-min averaging of high frequency database. This process, illustrated in Figure 2, allows for significant reduction of data amount and allows for data exchange with the use of a contemporary ship satellite communication system. Data measured directly onboard are supplemented additionally by weather data with the use of ship weather service. Weather data are interpolated along ship track and integrated with data measured onboard. 

In order to secure communication against unauthorized access, data package is encrypted with use of Rivesta-Shamira-Adlemana (RSA) and Advanced Encryption Standard (AES) algorithms. Data sent from the vessel are stored in cloud server and can be accessed by the owner of the technical office (usually superintendent or fleet manager) for analyses and comparison against the fleet.

**Figure 3:**
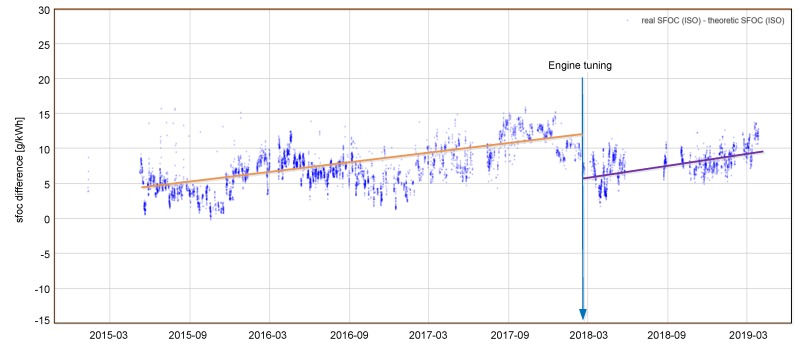
Main engine specific fuel oil consumption (SFOC) trend with visible gradual deterioration of engine performance [14]. (Courtesy of Enamor Ltd.).

Based on collected data, the ship performance monitoring system can be used for number of standard functions:

- Data reporting by aggregation, filtering and data grouping with respect to standard (noon, trip, Ship Energy Efficiency Management Plans (SEEMP), Monitoring, Reporting, Verification (MRV) [12], Data Collection System (DCS) for fuel oil consumption of ships) [13] and user defined reports;
- Faults detection in signal sources (sensors’ faults or connections’ faults) in order to maintain data access;
- Non-optimum operation warnings—system continuously evaluates selected signals according to specialized algorithms in order to recognize situations when vessel or her subsystems are operated in inefficient manner (e.g., concurrent operation of auxiliary engines at low output which can realized with lower number of units);
- Subsystems’ operational performance trending—in order to monitor subsystem performance deterioration and plan maintenance actions (e.g., hull fouling trend or monitoring of main engine specific fuel oil consumption (SFOC) trend as presented in Figure 3 [14]. In the monitored time period, an increased value of SFOC index was observed. In order to evaluate main engine performance, the system computes actual SFOC based on momentary fuel consumption and main engine load. Based on additional data collected by the system, actual SFOC is corrected to reference conditions [12,15,16]. In parallel, theoretical SFOC in reference conditions is calculated based on data specific for the engine, usually based on results of factory acceptance tests. Finally, difference between actual and theoretical SFOC is plotted as the time trend allowing for detection of gradual engine wear or improper engine tuning);
- Operational optimization—based on actual data and reference model [11] system recommends optimum parameters of operation (e.g., optimum trim with respect to ship draught and speed through the water).

Figure 3 presents typical SFOC analyses based on measurements taken onboard during vessel operation. It is visible that data collection is affected by considerable scatter. The reason for this is two-fold: an important factor influencing data scatter is measurement accuracy, secondly, the quality of reference SFOC model also influences results. First factor results in conditions of measurement onboard the ship during her operation. Harsh environmental conditions of ship engine room imply that measurement equipment usually compromise accuracy in order to get required robustness. Especially at low engine load where both fuel flow and shaft power meters operate at low rate, measurement errors are higher. As far as reference SFOC, it is usually obtained during engine shop tests where due to time constraints, only few (sometimes one) load points are exercised. In order to obtain a reference model for complete range of engine, loads interpolation and extrapolation techniques are used which introduce additional error. Despite obvious limitations of this approach, appropriate data processing in long term trending allows for taking practical conclusions. Linearization of time trend (indicated by orange and magenta lines in Figure 3) allows for making predictions of required engine maintenance and thus allowing for better planning. Correlation of rapid SFOC changes with engine tuning actions performed by crew in February 2018 (denoted on Figure 3 by a vertical arrow) in the case of electronically controlled engines allows for evaluation of tuning quality and its impact on engine performance. In this particular case, tuning resulted in drop of specific fuel consumption by ~6 g/kWh.

### 3.2. Measurement of the Operational Parameters of Main Engine

Main engine can be monitored based on general output or detailed signals describing engine operation performance such as cylinder combustion pressure. The latter requires special equipment since very high resolution of signal recording is required. In order to reproduce dynamics of combustion process, angular resolution of 0.1 degree of crankshaft position must be provided. For this purpose, FPGA processing unit in real time is employed. Data acquisition of following analog signals of engine operation are executed:

- Crankshaft angular position;
- Combustion pressure;
- Exhaust valve position (EVP);
- Injection quantity sensor position (for selected engines);
- Gas admission valves (GAV) positions (for selected engines).

**Figure 4:**
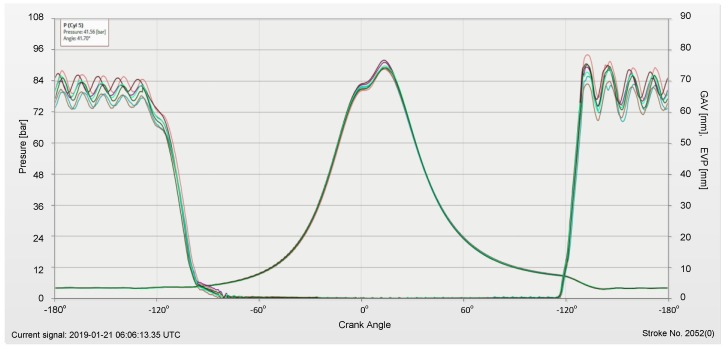
Combustion pressure monitoring of main engine [14]. (Courtesy of Enamor Ltd.).

The above data are post-processed and usually depicted in reference to cylinder top dead position as shown on Figure 4, in exemplary combustion pressure process monitoring.

**Figure 5:**
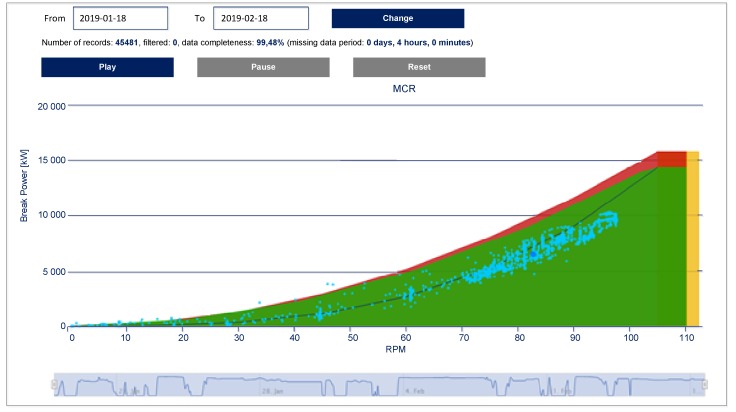
Main engine (ME) layout with actual operation points for one-month ship operation provided by SeaPerformer system; the records below the main engine layout present momentary changes of RPM on the ME shaft in the above-defined period (Courtesy of Enamor Ltd.).

Main engine general output may be monitored with use of shaft torque meter [14]. A good illustration of such a kind of measurement is propulsion control assistance system ETNP-10 for optimization of ship operation according to operation area of the main engine, being a part of information provided by SeaPerformer system, is depicted in Figure 5 [14]. Both cases actual engine load and rotational speed (dots) are presented in reference to so called engine layout or engine operational envelope. Engine layout presents standard operation area (green) over-speed area (yellow) and over-load area (red). Clear color indication allows ship crew to monitor engine operation and prevent conditions which may results in fault. As an extension to ETNP-10 user interface visualization which is devoted to actual engine conditions, SeaPerformer system may be used for engine monitoring in longer time range. The SeaPerformer system measures the torque on shaft and revolution (RPM- Revolution Per Minute) of the main engine. Other parameters like power, fuel consumption, energy and ship’s speed are calculated.

Based on the state-of-the-art [5,17,18], measurement of torque can be done with use of measurement of the torsion angle φ of propeller shaft during running of engine. Different torque measurement methods can be applied. In many methods, strain gauges as the sensors are used [5]. This solution requires the strain gauges to be placed directly on the rotating shaft and additional measurement of RPM should be performed. The strain gauges are configured in bridge system equipped with slip rings and brush contact devices. The strain gauges bridge is fed through the two slip rings and output signal taken from the bridge diagonal is also provided with two slip rings. The all slip rings are fitted on the rotating shaft and they provide a continuous electrical connection through brushes on stationary contacts. At the same time, slip ring and brush contact solution are important disadvantages of this method.

**Figure 6:**
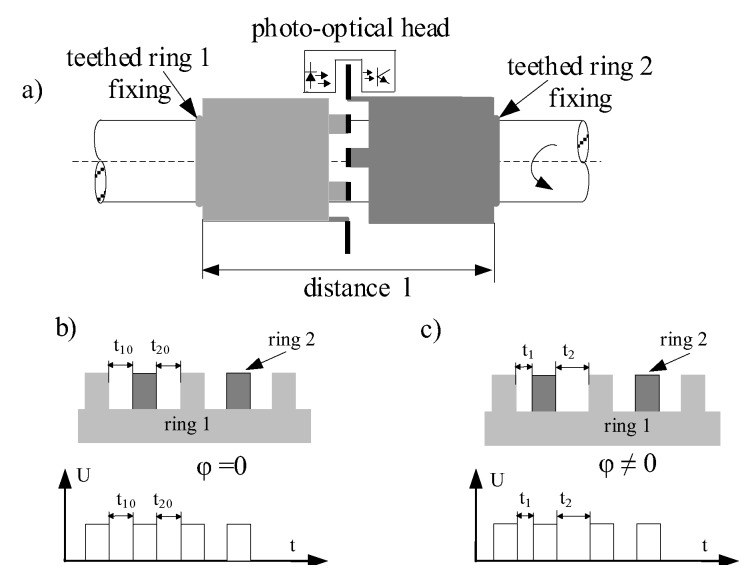
Photo-optical method for torque measurement, (**a**) concept of torque measurement, (**b**) relation between teeth of two rings and electrical signal for torsion angle φ = 0, (**c**) relation between teeth of two rings and electrical signal for torsion angle φ ≠ 0.

The other solution, free of aforementioned weaknesses, is based on the photo-optical method [17,18,19] (Figure 6), which uses two specially designed teethed rings. Each ring is fixed separately in same distance “l” on the shaft, but the teeth are placed in the same plane which is presented in Figure 6a. Such a solution gives possibility for use of only one photo-optical detector. The relation between teethed rings is proportional to the torsion angle of the shaft. In this method measurement of RPM by additional sensor is not required. In the case when the shaft is without load, the torsion angle φ = 0. This situation is presented in Figure 6b. Electric signal received from photo-optical detector shows that pulse period T_1_ and T_2_ are equal. When the load of the shaft occurs, the torsion angle is higher than 0. In such a situation, presented in Figure 6c, the periods of electrical signal are different and they are proportional to the measured value of torque M according to the formula:

(1) $$M=\frac{k_{T}\left(t_{1}-t_{2}\right)}{n}$$

where k_T_—coefficient depends on construction of shaft and teethed wheels, n—revolution, t_1_, t_2_—time of pulses received from photo-optical detector.

**Figure 7:**
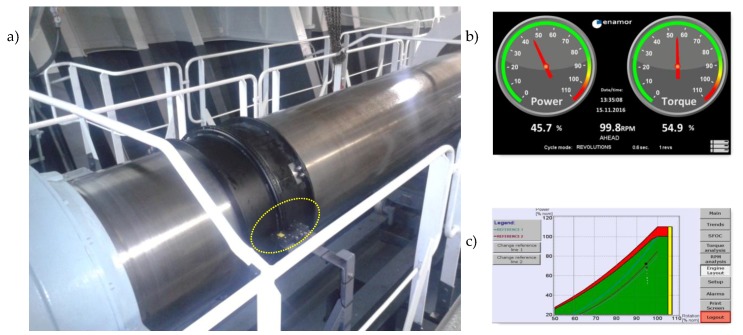
Propulsion control assistance system ETNP-10, where position of photo-optical detector is marked by ellipse, (**a**) two teeth rings installed on propeller shaft with photo-optical detector, (**b**) example of measurements, (**c**) actual engine load based on engine layout (Courtesy of Enamor Ltd.).

The method was developed [17,18] and patented in GMU [19] as well as implemented on many ships in cooperation with ENAMOR Ltd. company. Contribution of GMU in state-of-the-art development consisted of elaboration of a new solution of torque meter related transmitter [19]. A novelty of the transmitter solution [19] is based on special construction including two mutually advantageous elements, placed on the rotating shaft and each of them consist of the spacer pipe (distance sleeve) and measuring ring with evenly and radially distributed at the circumference adequate teeth. An advantage of this transmitter construction is use of only one photo-optical detector, that limits the influence of changeable conditions and work parameters of transmitter and electronic sets of photo-optical detector circuit on measurement accuracy. The validation tests of the propulsion control assistance system ETNP-10 type have executed by Research and Development Company ENAMOR Ltd. [14]. The investigated system installed on propeller shaft is presented in Figure 7 [14]. Figure 7a shows two teeth rings with photo-optical detector that are installed on propeller. The local human-machine interface presents current measurement parameters (Figure 7b) or engine load based on engine layout (Figure 7c).

### 3.3. Temperature Measurements in the Ship’s Hazardous Areas

Hazardous areas occur on almost all the ships. However, in certain ships, such as tankers (crude oil, chemicals, gas), mobile oil rigs or other offshore ships the hazardous areas are common. Due to technical conditions, the measurement of different parameters has to be made in the hazardous areas. 

Hazardous areas are the places where the risk of appearing mixture of flammable materials with oxygen from air might occur [20]. According to International Electrotechnical Committee (IEC) approach hazardous areas are divided into three zones for gas hazard: zone 0, zone 1, zone 2 and three zones for dust hazard: zone 20, zone 21 and zone 22. Depending on the zone, adequate explosion-proof equipment should be used. 

The IEC approach is commonly accepted by the majority of state maritime administrations and maritime classification societies [21,22,23].

Electrical equipment placed in the hazardous areas can be protected by different methods. For measurement and control, intrinsically safe solution “Ex i…” is the most adequate explosion-proof protection [24,25].

**Figure 8:**
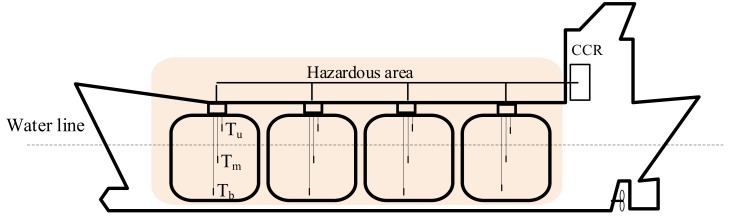
The temperature measurement of liquefied gas in tanks, CCR—Cargo Control Room, T_b_, T_m_—sensor in bottom and middle of tank to measure temperature of liquefied gas, T_u_—upper sensor to measure temperature of vapors.

In case of long distance measurement or control lines, which are typical on ships especially for deck monitoring and control, a special approach has to be used in comparison to the standard solution. The basic assumption of intrinsically safe solution is that inside of the intrinsically safe circuit, the accumulated electrical energy is so low that in the case of its realization in a form of a spark or a thermal effect, it is incapable of igniting surrounding explosive mixture. To ensure this, specially certified equipment has to be used inside the hazardous area and additional, also certified, associated apparatus has to be used and placed in the safe area. When both are connected by cables, this creates an intrinsically safe system [19]. It is worth underlining that in comparison to a standard solution, an associated apparatus is in fact an additional equipment connected to the measurement line. The associated apparatus is connected between intrinsically safe equipment placed in the hazardous area and the standard monitoring system placed also in the safe area. It can be a source of some limitations or even additional measurement errors. As an illustration of the discussed problem, the measurement of temperature in tanks of gas tanker is presented. The general scheme of the tanks placement and related temperature measurement is shown in Figure 8. 

#### 3.3.1. Accuracy Aspects of Temperature Measurement Line

**Figure 9:**
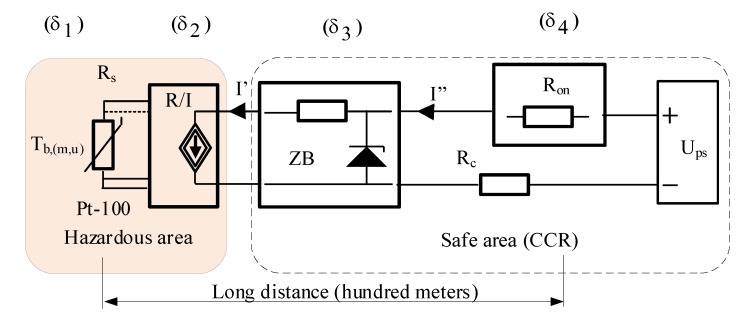
Functional diagram of temperature measurement line, where: R_s_—resistance of Pt-100 sensor, R/I—transmitter-source of current 4–20 mA sink type, ZB—Zenner barrier as the associated apparatus, R_on_—resistance of monitoring system, R_c_—equivalent resistance of cables, U_ps_—source of DC voltage, δ_1_, δ_2_, δ_3_, δ_4_ are relative errors.

Measurement of temperature can be performed in different ways [3,4], but platinum thermo-resistance Pt-100 is the most popular sensor for that. A predominant role of this kind of sensors results from the particular environmental and constructional conditions related to ship tanks, as well as with usually measured negative temperature range. Because of the long distance between measurement temperature point inside tanks and monitoring system placed in CCR, it is common to use a transmitter which converts resistance of a sensor to a standard current value. In our example, the two wires 4–20 mA standard is the most convenient to use [26]. The functional diagram of the discussed measurement line is presented in Figure 9.

In the analyzed case, combined standard uncertainty is based only on the uncertainty of B type and it is described by the formula:

(2) $$u_{c}=\left(\sqrt{\frac{1}{3}\left[{\left(\delta_{1}\right)}^{2}+{\left(\delta_{2}\right)}^{2}+{\left(\delta_{3}\right)}^{2}+{\left(\delta_{4}\right)}^{2}\right]}\right)\left(T_{2}-T_{1}\right)$$

where δ_1_, δ_2_, δ_3_, δ_4_ are relative errors of Pt-100 sensor, transmitter, Zenner barrier and monitoring system itself, T_1_, T_2_ are min and max of the temperature span.

For the real data δ_1_ = 1.6 × 10^−3^, δ_2_ = 3 × 10^−3^, δ_3_ = 1 × 10^−5^, δ_4_ = 2 × 10^−3^, T_1_ = −120 °C, T_2_ = 120 °C and related value of uncertainty corresponds to u_c_ = 0.57 °C. A set of value for liquefied gas of tanks corresponds to −90 °C. Accuracy of the temperature measurement in tanks illustrated in Figure 8 plays a fundamental role in estimation of the transported cargo volume and finally the transport cost and price of the gas. Therefore, all disturbing circumstances, causing additional errors in the process of temperature measurement, are very important. 

**Figure 10:**
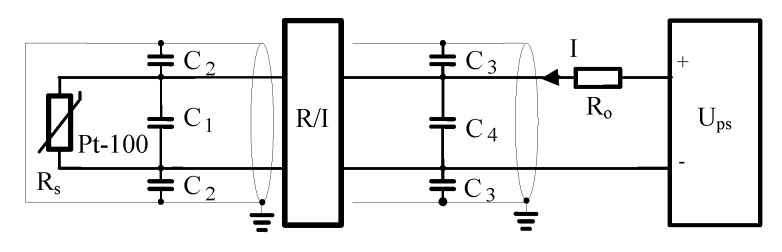
Equivalent electrical diagram of the temperature measurement line, where: R_s_—resistance of Pt-100 sensor, R/I—transmitter 4–20 mA, R_o_—equivalent resistance of all resistances in the 4–20 mA current loop I, C_1,2,3,4_—equivalent capacities of Pt-100 sensor and connecting cables, U_ps_—source of DC voltage.

On top of that, an additional error was observed. This error was caused in the real measurement circuits by own capacities of long distances of sensors, screened cables, grounding and dynamic properties of a transmitter. In reality, all the elements of the measurement line connected together produce an active electrical circuit with positive feedback. The equivalent electrical diagram of such temperature measurement line is presented in Figure 10.

**Figure 11:**
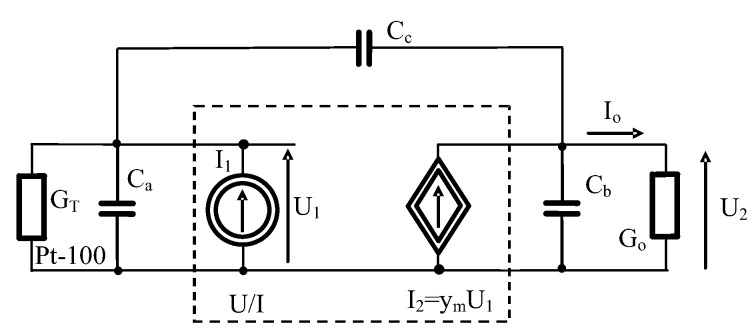
Small signal model of the temperature measurement line, where: G_T_—conductivity of Pt-100 sensor, U/I—transmitter 4–20 mA, G_o_—equivalent conductance of all resistances in the 4–20 mA current loop I_o_, C_a,b,c_—equivalent capacities of Pt-100 sensor and connecting cables, y_m_—transadmittance of transmitter R/I.

In order to analyze the above circuit, a small signal model of the measurement line is presented in Figure 11. Dynamic properties of transmitter R/I should be defined in the model. It is worth mentioning that transmitter R/I is the same as converter U/I with internal source of current I_1_ in Figure 11. Generally, the dynamic properties of the transducers are often omitted due to the fact that changes in the measured temperatures are relatively slow, especially when the industrial Pt-100 sensors are used. 

Actually, electrical dynamic properties of transmitter should be taken under consideration. They are described by transadmittance y_m_. Most often transmitters R/I are first-order inertial elements but sometimes they can be second-order oscillation elements. 

#### 3.3.2. Real Measurement Line with Transmitter as First-Order Inertial Element

For transmitters of first-order inertial type the transconductance y_m_

(3) $$y_{m}=g_{m}\frac{\omega_{2}}{s+\omega_{2}}$$

where g_m_—transconductance depends on measured range of temperature, ω_2_—3 dB frequency.

An analysis of stability of the circuit presented in Figure 7 was done for the set of real parameters given in Table 1 [1]. 

While performing computer simulations for the parameter values presented in Table 1, it was shown that the temperature measurement line with first-order inertial transmitter in special condition can work in an unstable manner and as a result an alternating current can occur.

The tendency to unstable working rises when the range is relatively small. That case is theoretical rather than practical because oscillating condition is fulfilled only for boundary conditions.

#### 3.3.3. Real Measurement Line with Transmitter as Second-Order Inertial Oscillation Element 

For transmitters of second-order oscillation inertial type, the transconductance y_m_ is given by the following formula

(4) $$y_{m}=g_{m}\frac{\omega_{o}^{2}}{s^{2}+2{\zeta \omega }_{o}+\omega_{o}^{2}}$$

where g_m_—transconductance depends on measured range of temperature, ω_o_—natural frequency, ζ—damping ratio. 

The analysis of stability of the circuit presented in Figure 11 was done for the set of real parameters given in Table 2 [1].

Computer simulations performed for parameters given in Table 2 have shown that temperature measured line with the oscillating second-order inertial transmitter can be very unstable. As a result, the theoretically direct current I_o_ is disturbed by alternating part. Further detailed research has shown that the frequency and shape of the additional alternating current depends not only on measured range but also strongly on value of measured temperature (T_o_) and equivalent load conductance (G_o_). 

**Figure 12:**
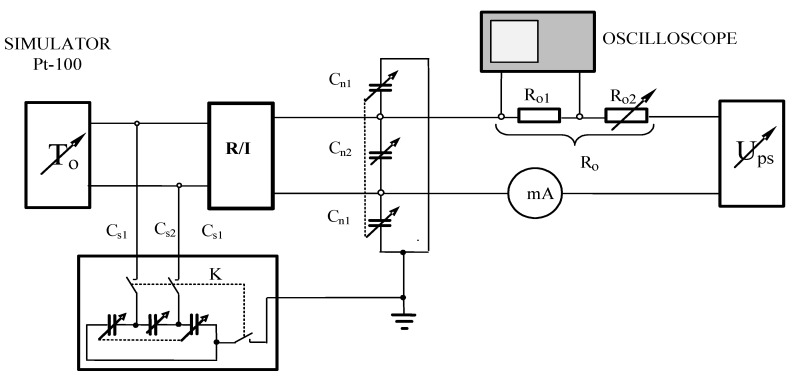
Laboratory experimental circuit of temperature measurement line with the possibility of taking account capacities of the Pt-100 sensor and connecting cables, where: R/I—second-order inertial transmitter, mA—DC ammeter, U_ps_—source of voltage, Ro—equivalent of all resistances in the 4–20 mA current loop, C_s1,2_—equivalents of own capacities of Pt-100 sensor, C_n1,2_—equivalents of own capacities of cables, K- switch.

The laboratory experiments of temperature measurement line with the oscillating second-order inertial transmitter R/I presented in Figure 12 were also performed and completely confirmed theoretical considerations. 

**Figure 13:**
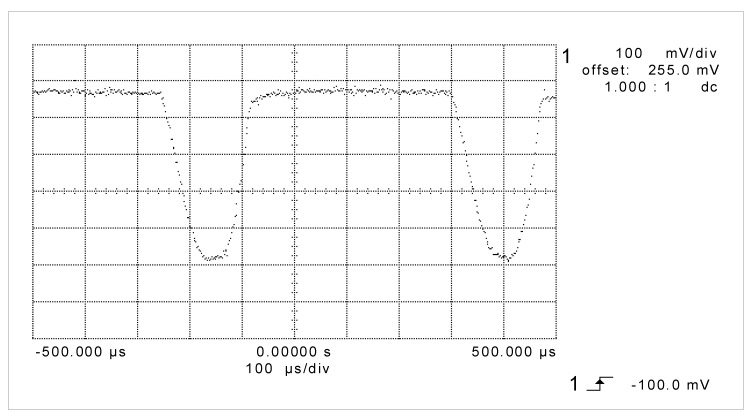
Exemplary of screenshot for a transmitter with a span ΔT = T_max_ − T_min_ = 60 − (−30) = 90 °C at measured temperature T_o_ = 50 °C for equivalent load resistance R_o_ = 600 Ω.

The experiments were performed for different simulated temperatures from measurement range of transmitter R/I (from −30 °C to 60 °C) and for different value of R_o_ for two cases. The first one where a capacitive feedback was broken (switch K-off position) and the second case where the own capacities of sensor by switch K in position on were taken into consideration. In the second case, the capacitive feedback was rebuilt. In both cases for the same simulated temperature, the current was measured by DC ammeter. While the capacitive feedback was done, sometimes the alternating current was observed by oscilloscope. An example of one such screenshot was presented in Figure 13 for a transmitter with a span ΔT = T_max_ − T_min_ = 60 − (−30) = 90 °C at measured temperature T_o_ = 50 °C for equivalent load resistance R_o_ = 600 Ω.

In Figure 13 an unexpected alternating current i(t) disturbed a correct temperature measurement procedure was presented. Actually, the shape of the alternating current depends on the measured temperatures. From that, a mean value of measured current I_m1_ by DC ammeter differs from measured value I_m2_ when only DC current appears while the capacitive feedback is off.

**Figure 14:**
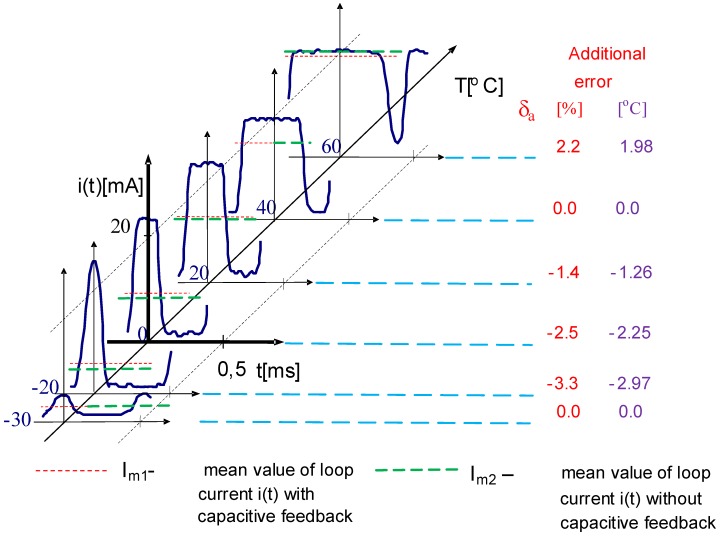
Graphical illustration of influence of unexpected alternating current on a measurement error.

To explain the nature of unexpected additional error while measuring temperature in the discussed cases, a collected set of alternating currents observed for different measured temperatures was presented in Figure 14. For legibility of the picture for a few selected temperatures, only a significant portion of the current waveforms has been included. For each graph, mean values for bath cases were pointed out. It is worth emphasizing that mean value of current I_m2_ depends on shape of current, which can be higher, lower or equal to the mean value of DC current in the case of lack of capacities feedback. Actually, that is observed by DC indicator as additional relative error $\delta_{a}$ of measured temperature and can be calculated according to the following formula:

(5) $$\delta_{a}=\frac{\left(I_{m2}{-I}_{m1}\right)\left[\mathrm{mA}\right]}{16\;\left[\mathrm{mA}\right]}\cdot 100\left[\%\right]$$

where value of 16 mA results from the 4–20 mA standard current transmitter.

To calculate the absolute value, simply multiply the relative error by the measuring range, which was mentioned above, in the discussed case is ΔT = 90 °C. The adequate values of additional absolute errors were presented in Figure 14 [27].

As a result, a new error appeared, and what is worse, that error was not observed during commissioning procedures while using only resistance simulator sensor.

To avoid such situations, in GMU there was patented an impedance simulator of Pt-100 sensors [28] introducing a new concept. According to this, during commissioning it is possible to take into account also the sensor’s own capacities. This solution improves significantly the quality and reliability of commissioning, and in consequence accuracy of evaluation of the quantity of transported liquefied gas.

## 4. Conclusions

In many cases, ship measurement methods require individual and specific approach, because of the marine environment influence. Such affecting factors like vibration, ship rolling and pitching, temperature, humidity, salinity or changeable weather conditions must be taken into account. In this context, well known currently existing and applied measurement methods and devices should be completed and developed. Two case studies, addressed to operational parameters of main engine and temperature measurement of liquefied gas in ship tanks, are shortly described from the perspective of a new approach to analysis of their operation in specific conditions, which are vibrating environment and hazardous area, respectively. This new approach is presented with stressing proposed novelties against the state-of-the-art and in relation to the operational parameters of main engine in ship power plant consists in, that:

- not only traditionally measured quantities on the main engine shaft like torque and RPM are measured but also some other main engine characteristic quantities, related to crankshaft angular position, combustion pressure, exhaust valve position, injection quantity sensor position and gas admission valve position are taken into account,
- in the presented photo-optical method based on the measurement of the torsion angle of the shaft, a measurement of RPM by additional sensor is not required, contrary to the method based on strain gauges. Moreover, presented photo-optical method is free of slip rings and brush contact devices, which are a serious maintenance problem in previously applied torque measurement method,
- described photo-optical method, aided by new solution of torque meter related transmitter gives possibility for use of only one photo-optical detector and at the same time the influence of changeable conditions and work parameters of transmitter and electronic sets of photo-optical detector circuit on measurement accuracy is significantly reduced comparing with other previously used solutions equipped with two photo-optical detectors,
- extended application of the aforementioned photo-optical method in SeaPerformer system enables not only measurement the torque on shaft and revolution (RPM) of the main engine, but also a calculation of other parameters like power, fuel consumption, energy and propeller slip. In consequence, also monitoring of main engine specific oil consumption (SFOC) trend is executed. Aforementioned monitoring is supported by data acquisition of engine operation signals related to crankshaft angus or position, combustion pressure, exhaust valve position (EVP), injection quantity sensor position and gas admission valve (GAV) position respectively,
- Next, in wider perspective, engine performance evaluation based on momentary fuel consumption analysis and main engine load is carried out. Monitoring of the above listed operational parameters of main engine aided by a new class of systems like SeaPerformer gives possibility for operational optimization of main engine exploitation and constitutes a new perspective comparing with hitherto used solutions in considered field of research.

On the other hand, the new approach of analysis in relation to the temperature measurement in ship tanks, with placing emphasis on presented novelties consists in, that:

- Temperature measurement in ship’s hazardous areas covers not only use of intrinsically safe solutions for design of temperature measurement line, but also takes into account the additional measurement errors caused in the real measurement circuits by own capacities of long distances of sensors (predominant solutions are based on the Pt-100 sensors), screened cables, grounding and dynamic properties of a transmitter.
- Predominant solutions of sensor due to specific conditions of temperature measurement in ship tanks are based on the Pt-100 elements applied in intrinsic safe version of the measurement line, often equipped with passive Zener Barrier; Passive Zener Barrier dependently of the circuit parameters may enhance additional measurement error and what is worse, that error was not observed during commissioning procedure while using only resistance simulator Pt-100 sensor,
- To avoid unexpected alternating current component disturbing a correct temperature measurement, there was patented a new impedance simulator of Pt-100 sensors,
- This new solution enables to take into account also the sensors own capacities and in consequence, to improve significantly the quality and reliability of commissioning.
- Finally, accuracy of evaluation of the quantity of transported liquefied gas (corresponding to cost of transport) is most reliable.

Future analyses, respectively in the two areas under consideration, will focus on:

- In the scope of operational parameters of the main engine in a ship power plant—on the further development and implementation of automatic detection of engine anomalies and failures and context-based information selection both in onboard and on-line applications,
- In the area of temperature measurement on ships—on carrying out an analysis of operating conditions, in which an additional temperature measurement error may occur, resulting from the dynamic properties of electronic measuring line elements, where the sensor is a thermocouple. The different types of T/C converters and the impact of the intrinsically safe solution will be taken into account.

## Figures and Tables

**Table 1:** Parameters of real temperature measurement line for first-order inertial transmitter.

Parameter [unit]	Value/Range	Parameter [unit]	Value/Range
C_a_ [nF]	4.5	G_o_ [mS]	2
C_BS_ [nF]	8	g_m_ [S]	0.043–0.83
C_c_ [nF]	1.7	ΔT^2^ [°C]	50–1050
G_T_ [mS]	8.34	ω_2_ [rd/s]	14,700
T_o_^1^ [°C]	50	-	-

^1^ T_o_ measured temperature. ^2^ ΔT temperature measured span.

**Table 2:** Parameters of real temperature measurement line for oscillating second-order inertial transmitter.

Parameter [unit]	Value/Range	Parameter [unit]	Value/Range
C_a_ [nF]	4.5	G_o_ [mS]	2
C_b_ [nF]	8	g_m_ [S]	0.043–0.83
C_c_ [nF]	1.7	ΔT [°C]	50–1050
G_T_ [mS]	10	ω_o_ [rd/s]	20,400
T_o_ [°C]	0	ζ	0.212
